# Versatile pattern generation of periodic, high aspect ratio Si nanostructure arrays with sub-50-nm resolution on a wafer scale

**DOI:** 10.1186/1556-276X-8-506

**Published:** 2013-12-01

**Authors:** Jian-Wei Ho, Qixun Wee, Jarrett Dumond, Andrew Tay, Soo-Jin Chua

**Affiliations:** 1 NUS Graduate School for Integrative Sciences and Engineering, National University of Singapore, Centre for Life Sciences, #05-01, 28 Medical Drive, Singapore 117456, Singapore; 2 Centre for Optoelectronics, Department of Electrical and Computer Engineering, National University of Singapore, Block E3 02-07, Engineering Drive 3, Singapore 119260, Singapore; 3 Institute of Materials Research and Engineering, Agency for Science Technology and Research (A*STAR), 3 Research Link, Singapore 117602, Singapore; 4 Singapore-MIT Alliance, National University of Singapore, Block E4 04-10, 4 Engineering Drive 3, Singapore 117576, Singapore; 5 Department of Mechanical Engineering, National University of Singapore, Block EA 07-08, 9 Engineering Drive 1, Singapore 117576, Singapore; 6 Singapore-MIT Alliance for Research and Technology Center, 1 CREATE Way, #10-01 CREATE Tower, Singapore 138602, Singapore

**Keywords:** Sub-50-nm resolution, User-defined patterns, Wafer scale, Non-porous, Si nanostructures, Step-and-repeat nanoimprint lithography, Metal-catalyzed electroless etching, 81.16.Hc; 81.16.Nd; 81.05.Cy

## Abstract

We report on a method of fabricating variable patterns of periodic, high aspect ratio silicon nanostructures with sub-50-nm resolution on a wafer scale. The approach marries step-and-repeat nanoimprint lithography (NIL) and metal-catalyzed electroless etching (MCEE), enabling near perfectly ordered Si nanostructure arrays of user-defined patterns to be controllably and rapidly generated on a wafer scale. Periodic features possessing circular, hexagonal, and rectangular cross-sections with lateral dimensions down to sub-50 nm, in hexagonal or square array configurations and high array packing densities up to 5.13 × 10^7^ structures/mm^2^ not achievable by conventional UV photolithography are fabricated using this top-down approach. By suitably tuning the duration of catalytic etching, variable aspect ratio Si nanostructures can be formed. As the etched Si pattern depends largely on the NIL mould which is patterned by electron beam lithography (EBL), the technique can be used to form patterns not possible with self-assembly methods, nanosphere, and interference lithography for replication on a wafer scale. Good chemical resistance of the nanoimprinted mask and adhesion to the Si substrate facilitate good pattern transfer and preserve the smooth top surface morphology of the Si nanostructures as shown in TEM. This approach is suitable for generating Si nanostructures of controlled dimensions and patterns, with high aspect ratio on a wafer level suitable for semiconductor device production.

## Background

Silicon nanostructures have unique optical, electrical, and thermoelectric properties not observed in its bulk embodiment. The advantages conferred by these traits have seen Si nanostructures being applied in nanoelectronics for transistor miniaturization [1-3], photovoltaics for exceptional light trapping [4-6], and photodetection for ultrahigh photoresponsivity [7]. Si nanostructures such as Si nanowires (SiNWs) have also enabled ultra-sensitivity to be realized in chemical and biological sensing [8], efficient thermoelectric performance [9], enhanced performance in Li-ion batteries [10], and nanocapacitor arrays [11].

Successful realization of Si-nanostructured devices on a manufacturing scale, however, requires practical techniques of producing the nanostructures with controlled dimensions, patterns, crystalline structures, and electronic qualities. Metal-assisted chemical etching (MACE) or metal-catalyzed electroless etching (MCEE) is a simple technique first demonstrated by Peng et al., which can be used to generate high aspect ratio Si nanostructures [12,13]. In this manuscript, this technique is referred to as MCEE because this provides a more explicit description of the process. Sidewall inclination common in reactive ion etching (RIE) [14] and scalloping effects typical of deep reactive ion etching [15] are avoided in MCEE. The process does not require the complex precursors used in vapor-liquid-solid growth or chemical vapor deposition, and the expensive equipment of inductive coupled plasma-RIE or DRIE. Properties such as doping level and type, crystal orientation, and quality are determined simply by the starting Si wafers.

Approaches combining nanoscale patterning techniques with MCEE have been reported. The combination allows more control over the order, diameter, and density of the Si nanostructures. This was demonstrated with nanosphere lithography which is based on the self-assembly of a monolayer of nanospheres (e.g., polystyrene [16] or silica [17]) into ordered hexagonal close-packed arrays. However, ordering of the nanospheres and the resulting Si nanostructures are limited to domains. Huang et al. employed an anodic aluminum oxide (AAO) template and a Cr/Au evaporation step to define the mask for catalytic etching to form SiNWs [18]. While this is a simple and cost-effective method, the positions of the nanostructures are limited to short-ranged hexagonal arrangements, and large-scale production will likely be hampered by inefficient AAO template transfer to the Si substrate. Lately, block copolymer lithography has been paired with MCEE to produce highly dense Si nanostructure arrays. But a distribution of dimensions exists, and ordered arrangement is limited to small areas [19].

In order to fabricate Si nanostructures with various array configurations, cross-sectional shapes, and perfect ordering over large areas, interference lithography (IL) in combination with MCEE has been employed by Choi et al. [20]. Si nanostructures with circular or square cross-sections and Si nanofin arrays can be readily obtained by tuning the IL exposure process. This is an attractive lithographic process that can be used to rapidly generate perfectly periodic patterns over large areas. Through this approach, SiNWs of sub-100-nm diameters have been achieved [21].

Despite the advantages of IL, the density and lateral dimension of Si nanostructures are ultimately limited by the wavelength of the incident light [20], an issue common with UV and DUV photolithographies. Furthermore, the cross-sectional shapes and array configurations are constrained to those permitted by interference. While advanced nanolithography techniques such as electron beam lithography (EBL) are capable of realizing feature dimensions down to a few nanometers, and are valuable tools in a research environment, they are not amenable to an industrial high-throughput manufacturing setting [22]. These limitations are circumvented with nanoimprint lithography (NIL) in which the mould pattern can be written by EBL and thus have excellent versatility in pattern design and resolution similar to EBL. Wafer-scale patterning can subsequently be achieved by direct large-area nanoimprinting [23,24] or through a stepper.

Recently, substrate conformal imprint lithography was used in combination with MCEE by Wang et al. to produce ordered arrays of elliptical nanopillars. Unfortunately, the generated nanostructures, of relatively large dimensions (several hundreds of nanometers), do not realize the high resolution potential offered by NIL and also exhibited a high degree of porosity [25]. A combinatory technique consisting of soft lithography, SiN_*x*_ deposition and etching, and MCEE has also been reported by Balasundaram et al. [26], but the elaborate procedure negates the simplicity of MCEE.

In this work, we employ a simple two-stage procedure consisting of step-and-repeat nanoimprint lithography (SRNIL) [27] with etch-resistant NIL resin chemistry, and optimized MCEE conditions to fabricate wafer-scale, near perfectly ordered, single crystalline, non-porous silicon nanostructures with controlled feature sizes down to sub-50 nm. Circular, hexagonal, and rectangular cross-sectional Si nanostructures in hexagonal or square array configurations with 150- or 300-nm periods (corresponding to array packing densities up to 5.13 × 10^7^ structures/mm^2^) and aspect ratios as high as 20:1 or more were produced using EBL-defined NIL pore-patterned moulds and MCEE. The results clearly illustrate the high resolution potential of NIL and deep-etching capabilities of MCEE. To our knowledge, this is the first demonstration of versatile pattern generation of near perfectly ordered Si nanostructures down to sub-50-nm feature sizes via SRNIL and MCEE on a wafer scale. This offers a simple and fast route towards semiconductor nanostructured device production.

## Methods

### Wafer-scale step-and-repeat nanoimprint lithography

**Figure 1:**
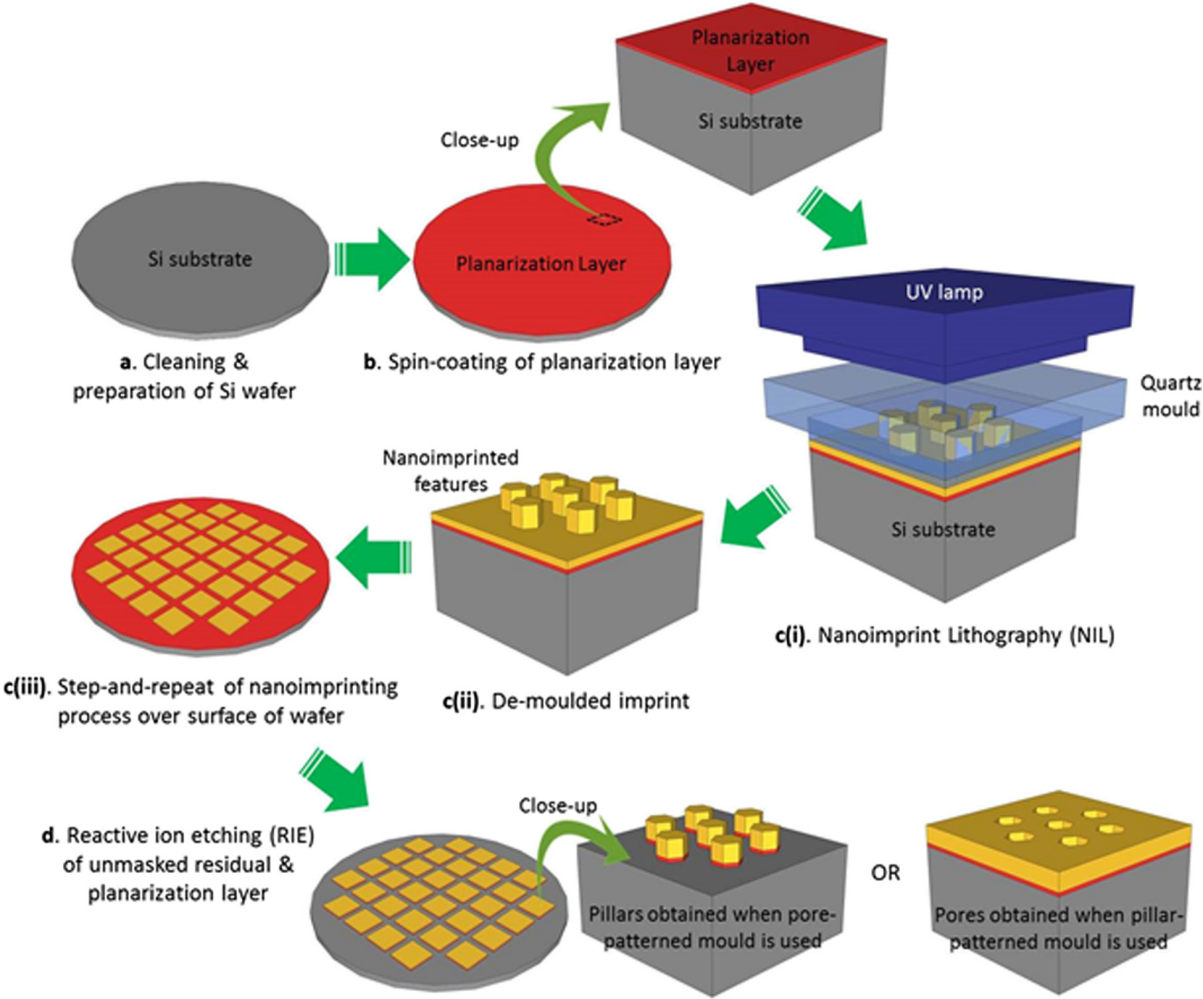
Schematic diagram illustrating steps involved in step-and-repeat nanoimprint lithography (SRNIL) to produce pillar- or pore-patterned nanoimprinted wafers.

Wafer-scale nanoimprinted samples were first generated via SRNIL. The experimental steps involved are schematically illustrated in Figure 1 and briefly described as follows: 4″ boron-doped, p-type Si(100) wafers (resistivity 10 to 20 Ω · cm) were thoroughly cleaned in boiling piranha solution, spin coated with an organic planarization layer (Transpin from Molecular Imprints, Inc., Austin, TX, USA), loaded into the SRNIL equipment, and leveled against a patterned quartz template/mould. For each target imprint area, nanoliter droplets of UV-curable, low-viscosity acrylate resist (MonoMat from Molecular Imprints, Inc.) were dispensed onto it and the quartz mould was brought into close proximity with the substrate, thus displacing the resist. This induced the resist to spread across the imprint field and fill up the mould relief via capillary action. A short exposure to UV light caused the polymerization of the monomers in the resist, after which the mould was separated from the substrate, leaving behind an inverse replica of the mould pattern. This UV nanoimprint process was optimized for full pattern transfer while minimizing the residual material at the base of the recessed features and maintaining its uniformity across the field. The optimized nanoimprint process was step-and-repeated over the surface of the wafer to achieve wafer-scale nanopatterning. The residual layer and underlying planarization layer were then removed by an oxygen reactive ion etching (RIE) process, thus exposing the underlying Si in these regions.

**Figure 2:**
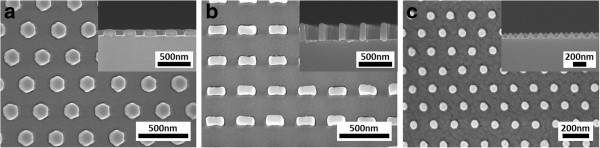
**SEM images of the nanoimprinted samples after RIE.** Inset shows the respective cross-sections. **(a)** 300-nm period hexagonal array of 180-nm (facet-to-facet) hexagonal pillars/studs, **(b)** 300-nm period square array of 200-nm × 100-nm rectangular pillars, and **(c)** 150-nm period hexagonal array of 50-nm diameter circular studs.

In this work, three different pore-patterned quartz moulds were employed, allowing the corresponding inverse patterns to be defined. The replicated patterns consist of (a) 300-nm period hexagonal array of 180-nm (facet-to-facet dimension) hexagonal pillars/studs, (b) 300-nm period square array of 200 nm × 100-nm rectangular pillars, and (c) 150-nm period hexagonal array of 50-nm diameter circular studs. By incorporating some degree of lateral etching in RIE after NIL to remove the residual material in the recessed regions, NIL pillars/studs can be narrowed, thereby providing some tunability in the dimensions of the NIL features. The patterns are shown in Figure 2a,b,c.

**Figure 3:**
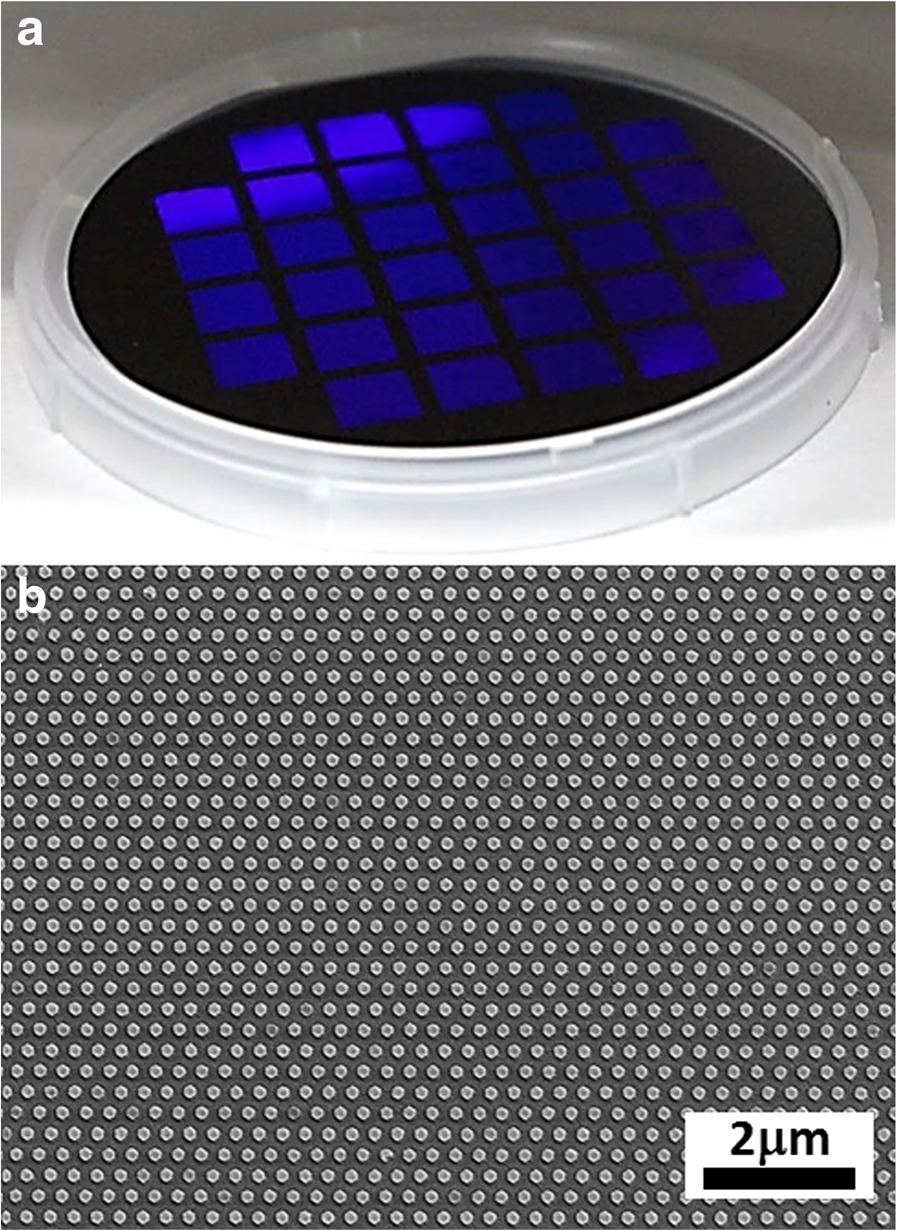
**Photograph of nanoimprinted 4″ Si wafer (a) and SEM image showing long-range order of corresponding nanostructures (b).** The wafer in **(a)**, produced by SRNIL, was deliberately tilted at an angle to bring out the violet-blue tinge arising from the optical diffraction caused by the highly ordered nanoimprinted hexagonal studs of 300-nm periodicity.

The patterned area in each 300-nm period mould is 10 mm × 10 mm, while that for the 150-nm period mould is 5 mm × 5 mm, enabling equal-sized imprints to be replicated over a wafer surface. An instance of wafer-level nanoimprinting by SRNIL is shown in Figure 3. In this case, 32 nanoimprinted fields were generated over the surface of a 4″ Si wafer. The street size between the fields can be varied to accommodate more or less fields. Furthermore, by virtue of the step-and-repeat mechanism, the NIL process can be extended for up to 8″ wafers.

### Metal-catalyzed electroless etching

The mechanism of MCEE is well discussed in literature and will not be described at length here [28]. Briefly, in a solution of HF and an oxidative agent, e.g., H_2_O_2_, of appropriate concentrations, regions of Si that are in contact with a noble metal, such as Au or Ag, are etched much faster than those regions without metal coverage. This phenomenon arises because the noble metal acts as a catalyst facilitating the local injection of holes into Si, resulting in its oxidation and subsequent removal by HF. The reaction is redox in nature and the metal ‘sinks’ into Si, creating an etched path. Therefore, by pre-patterning a noble metal layer on Si prior to immersion in HF/H_2_O_2_, patterned etched structures can be generated.

**Figure 4:**
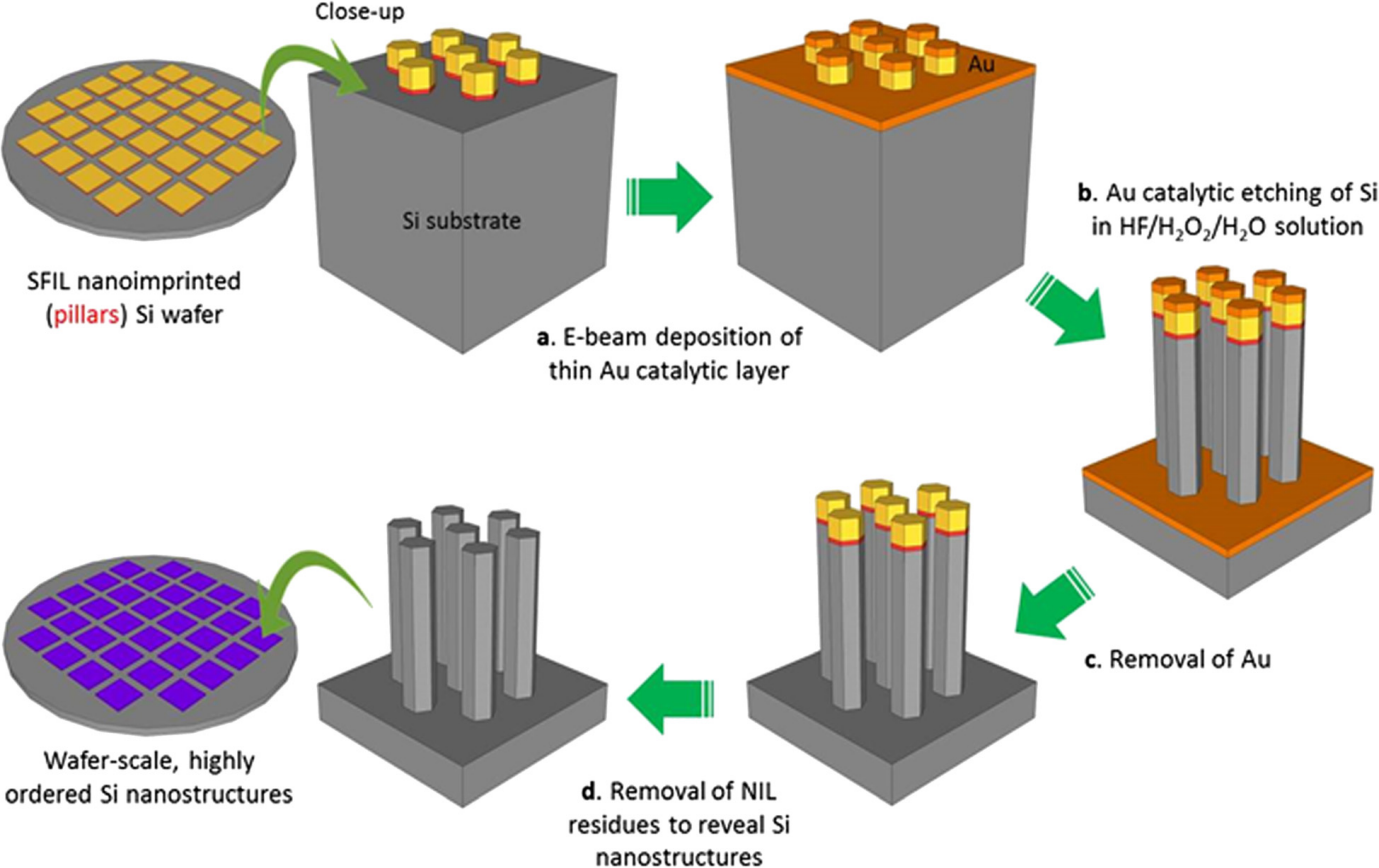
The generation of wafer scale, highly ordered Si nanostructures from a SRNIL nanoimprinted Si wafer via MCEE.

The steps leading up to MCEE for the stud-patterned wafers are described as follows and schematically shown in Figure 4. After the removal of the residual material at the recessed regions by RIE, a thin layer of Au (approximately 20-nm thick) acting as the catalyst was deposited by electron beam evaporation at a pressure of approximately 10^-6^ Torr. The wafer was then immersed in a solution of 4.6 M HF and 0.44 M H_2_O_2_ for the required period of time, after which the reaction was halted by rapid removal of the wafer from the chemical solution and subsequent immersion in deionized water. Next, the Au layer was removed in aqua regia at 70°C, and the NIL mask was stripped in boiling piranha solution to reveal the Si nanostructures.

## Results and discussion

**Figure 5:**
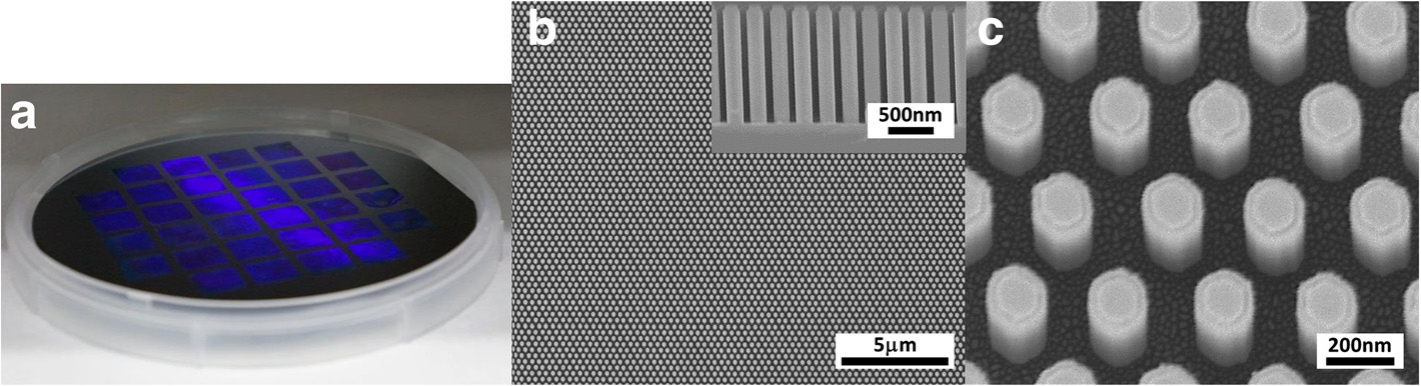
**Photograph and SEM images of wafer-scale Si nanostructures formed by the combined approach of SRNIL and MCEE. (a)** Photograph of a 4″ Si wafer consisting of 32 arrays of hexagonally ordered hexagonal Si nanopillars. **(b)** SEM image showing the hexagonal long-range order of the Si nanopillars. Inset shows the cross-sectional SEM image of the Si nanopillars showing the relatively straight sidewalls and NIL mask cap. **(c)** SEM plan view of the Si nanopillars (approximately 160-nm wide) showing the NIL mask cap on the top surface of each structure.

Figure 5a shows a 4″ Si wafer bearing 32 fields (each 10 mm × 10 mm) of hexagonal Si nanopillars in a hexagonal arrangement generated by the aforementioned approach. The near-perfect ordering of the Si nanopillars can be deduced from the optically diffracted violet-blue light when the wafer was tilted at an angle against a diffused white light source. The near-perfect long-range ordering is also observed in the SEM image of Figure 5b. Figure 5c shows the closed-up SEM plan view of the hexagonal Si nanopillars. The period of the nanopillars is 300 nm (corresponding to an area density of 1.28 × 10^7^ pillars/mm^2^) as defined by the nanoimprinting mould, while the lateral facet-to-facet dimensions is approximately 160 nm, a reduction from the approximately 180-nm pores in the NIL mould. The NIL mask which forms a cap over each nanopillar is visible in the SEM images demonstrating the resistance of the material to attack by the HF/H_2_O_2_ etching solution. The inset of Figure 5b shows the SEM cross-section of the Si nanopillars, revealing the etched profiles, straight sidewalls, and NIL mask caps. The height of the etched hexagonal Si nanostructures is approximately proportional to the etching duration, indicating a near-constant etch rate (approximately 320 nm/min). By varying the time of etching, the height of the structures can be adjusted, thus tuning the aspect ratio.

Molar concentrations of HF and H_2_O_2_, abbreviated as [HF] and [H_2_O_2_], respectively, other than that reported in this work (4.6 M HF and 0.44 M H_2_O_2_), have been employed in our experiments. However, it is found that 4.6 M HF and 0.44 M H_2_O_2_ are optimal for rapidly generating high aspect ratio Si nanostructures with sidewalls of low porosity. Similar concentrations have also been used by other works reported in the literature [18,20,21,29,30]. The influence of [HF] and [H_2_O_2_] in fabricating the Si nanostructures in MCEE has been discussed by Lianto [29] and Lianto et al. [31]. According to them, the porosity of the etched nanostructures is controlled by the concentration of excess electronic holes in Si. Since the flux and consumption of the electronic holes depend on [H_2_O_2_] and [HF], respectively, these are crucial in determining the structure of the etched bodies and the etch rate. Higher [H_2_O_2_] is correlated with increased porosity because the flux of the electronic holes injected into Si is higher, and more excess holes can diffuse from the catalyst to cause porosity in other regions of the Si nanostructures. A similar phenomenon has been observed in our experiments and by Wang et al. [25] where higher [H_2_O_2_] leads to increased sidewall roughness and structure porosity. However, even with increased [H_2_O_2_], etching occurs much faster in the regions of Si covered by the Au catalyst such that a large degree of anisotropy is maintained, albeit at the expense of greater sidewall roughness and porosity, especially near the top of the Si nanostructures. Conversely, a low [H_2_O_2_] is still insufficient to eliminate porosity in the Si nanostructures when [HF] is low, although it allows a slower, more controllable etch rate.

Increasing [HF] can significantly reduce the porosity of the sidewalls, while also increasing the etch rate [29]. Unfortunately, an excessively high [HF] leads to the increased evolution of H_2_ bubbles which can interfere with the spatial etch uniformity. As aforementioned, 4.6 M HF and 0.44 M H_2_O_2_ are chosen as an optimal combination. However, lower concentrations, possibly in similar relative molar ratios, may also be employed to provide a slower etch rate but with minimal porosity for the generation of lower aspect ratio Si nanostructures in MCEE. Hence, depending on the degree of nanoporosity and etch rate required, the concentration of the MCEE solution can be suitably tuned.

Due to the lack of an etch stop layer in MCEE, controlled halting of the wet etching process requires rapid removal of the wafer from the etching solution and subsequent immersion/rinsing in a bath of non-reacting dilution medium (deionized water in this case). This technique quenches the reaction, and good spatial control can be effected provided that the removal and immersion/rinsing steps can be executed in a much shorter time frame (approximately 1 s, in our case) relative to the total etch time. Considering the etch rate of approximately 320 nm/min, etch depths of several hundreds of nanometers to more than a micron can be achieved with low relative spatial etch depth variation, since the absolute difference in spatial etch depth represents only a small fraction of the height of the Si nanostructures. For shallower etch depths, a slower, more controlled etch rate would be recommended and can be achieved by lowering [HF] and [H_2_O_2_] but in suitable molar concentration ratios. Large-scale reproducibility in large wafers may require suitable engineering control methods such as large baths of deionized water under constant agitation or rapidly flowing deionized water for quenching of reaction and rinsing.

**Figure 6:**
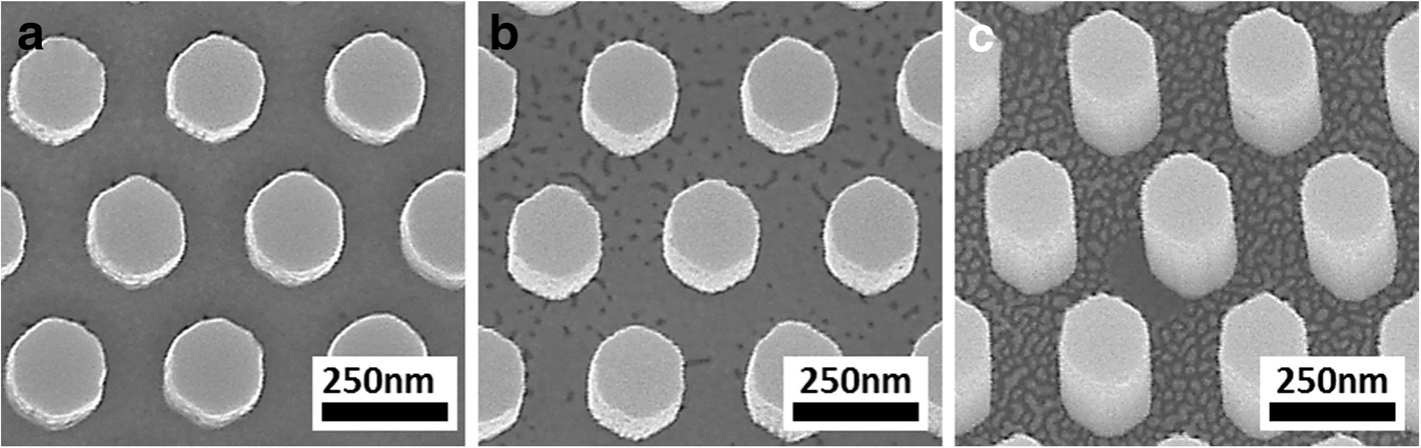
**Plan view SEM images of Si nanostructures after different etch durations with the NIL masks removed. (a)** 30-, **(b)** 60-, and **(c)** 180-s etch durations. The top surfaces of the nanostructures remain smooth after the process due to a good degree of protection offered by the NIL masking layer. This contrasts with the rougher sidewalls. Slight narrowing in the lateral dimensions of the Si nanostructures from approximately 180 nm to approximately 160 nm occurs when the etching duration is increased from 30 to 180 s. The fine lines or streaks observed in **(b)** and to a greater degree in **(c)** between the Si nanostructures are attributable to non-uniform gold coating of low-relief surfaces between higher structures prior to FESEM to reduce charging effects.

Unlike other reported Si nanostructures produced by metal-assisted chemical etching which sports a highly roughened top surface due to chemical attack, with the degree of roughening increasing with etch duration [16-18,20,21,28], our technique produces Si nanostructures with considerably smoother top surfaces. As shown in Figure 6, the top surface of the Si nanostructure remains well-defined and flat after MCEE and NIL mask removal. However, a slight narrowing of the hexagonal Si nanopillars (from approximately 180 nm to approximately 160 nm) occurs with increased duration of etching (from 30 to 180 s). This should be taken into consideration when fabricating Si nanostructures with low tolerance for dimensional deviations. While this lateral component of etching is much slower than the reaction occurring directly at the regions of Si covered by the Au catalyst, thus conferring a high degree of anisotropy to the MCEE process, it will nonetheless impose a limit to the maximum achievable aspect ratio. An aspect ratio as high as 20:1 has been obtained in our experiments, but the maximum value will likely be limited by dissolution of the Si nanowires [21]. Aspect ratios up to 220:1 have been achieved [19].

**Figure 7:**
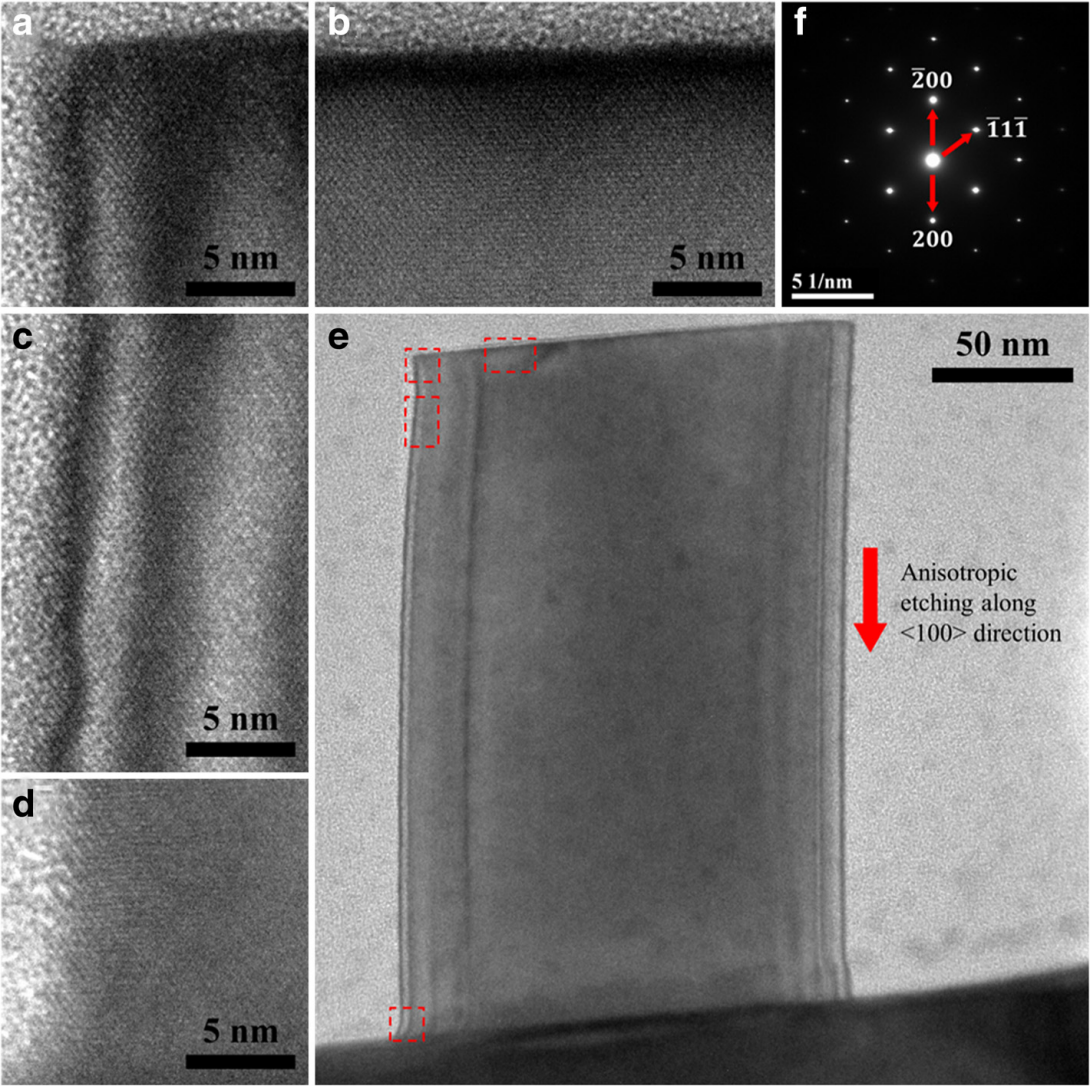
**HR-TEM images of metal-catalyzed electrolessly etched Si nanostructure (after a 60-s etch and removal of NIL mask). (a)** Top left corner. **(b)** Top surface. The well-defined and flat top interface is a consequence of the resistance of the NIL mask against chemical attack. **(c)** Left sidewall near the top surface. The etched sidewall shows a higher extent of surface roughness of about 3 nm due to attack by the HF/H_2_O solution. **(d)** Left sidewall towards base of nanostructure. Surface roughness is smaller due to shorter exposure to etching solution. **(e)** TEM image of the entire MCEE Si nanostructure. Red-outlined boxes show the locations of where the magnified HR-TEM images were taken. The etching proceeds preferentially along the <100 > direction. **(f)** The single crystal quality of the Si is evident from the SAED pattern.

While maintaining relatively low doping levels in the Si wafers (resistivity 10 to 20 Ω.cm) may play a contributory role in slowing the progress of porosity attack, the preservation of the smooth top surface is more likely linked to the use of the NIL mask. The latter is formed by the UV polymerization of a proprietary silicon-containing acrylate resist, the adhesion of which is strongly enhanced by the use of the planarization/primer layer. This is shown to be highly resistant to chemical attack by both acids and bases, with complete removal being effected by immersion in boiling piranha solution only. The NIL mask caps remain after MCEE and are shown in Figure 5b,c. The observations show that under our conditions of etching, the mask offers good protection to the Si surface against chemical attack by the HF/H_2_O_2_ etching solution. The integrity of the Si nanostructure is further shown in the high-resolution transmission electron microscopy (HR-TEM) images of Figure 7. A smooth morphology of the top surfaces (Figure 7a,b) is observed in contrast to the rougher sidewalls (Figure 7c,d). The preservation of the top surface can have potential device applications which are currently being explored.

As shown in the magnified TEM images of Figure 7c,d, the bottom of the Si nanostructure has smoother sidewalls than the top because of shorter exposure to the etching solution, and this is consistent with reports in literature [16,28]. The selected area electron diffraction (SAED) pattern in Figure 7f is obtained from near the tip of a single nanorod. The sharp and clear SAED pattern is typical of a single-crystal face-centered cubic material like silicon, observed in the (011) beam direction. No stray spots or elongation of spots is observed, indicating that high crystal quality is maintained after the etching. Figure 7 shows that MCEE occurs largely along the <100 > direction away from the top surface of the Si(100) wafer. The observed anisotropy of MCEE in Si is consistent with the reports in literature [16-18,20,21,28,32,33] and may be explained by the back-bond breaking theory [33,34]. Briefly, each atom on the (100) surface has only two back-bonds compared to three for that on the (110) and (111) surfaces, such that the former has a weaker back-bond strength. It is thus more easily removed during MCEE, and the etching occurs preferentially along the <100 > direction.

**Figure 8:**
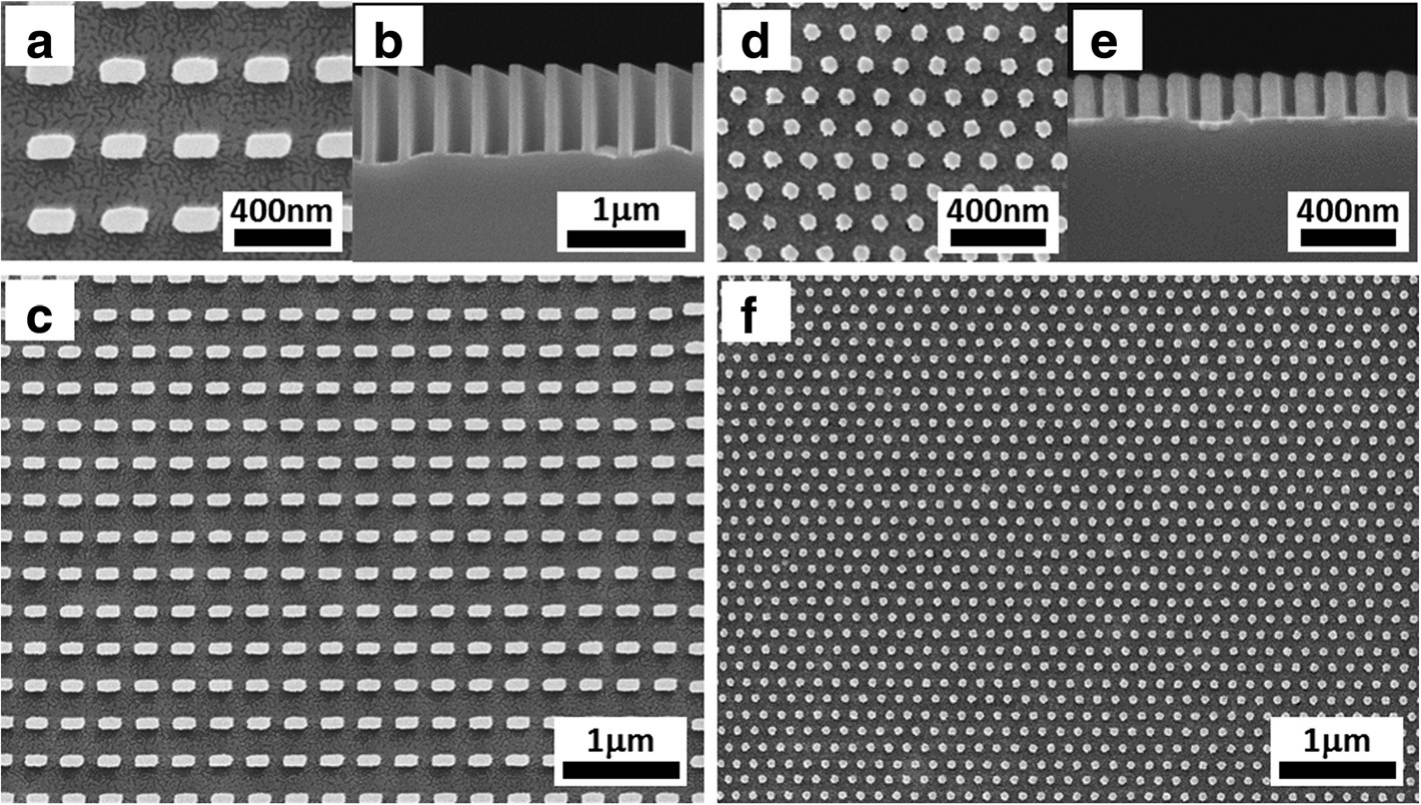
**SEM images of Si nanostructures generated by SRNIL and MCEE. (a**,**b**,**c)** Close-up, cross-section, and overview of a 300-nm period square array of 190 ± 3 nm by 95 ± 2 nm rectangular cross-section Si nanopillars. **(d**,**e**,**f)** Corresponding views of a 150-nm period hexagonal array of sub-50-nm (46 ± 2 nm) diameter cylindrical Si nanopillars.

Other SRNIL patterns may similarly be transferred into the underlying Si substrate by MCEE. Figure 8 shows the Si nanostructures (190 ± 3 nm by 95 ± 2 nm rectangular cross-section and 46 ± 2-nm diameter circular cross-section of pillars) generated from the patterns in Figure 2b,c. The results demonstrate that the array configurations are not restricted to hexagonal arrangement alone and may be extended to square arrays too. In addition, the Si nanostructures may take on other cross-sectional shapes such as rectangular or circular profiles with feature dimensions down to sub-50 nm. Aspect ratios up to 20:1 or more have been achieved, but the compliant Si nanowires have a tendency to adhere to each other due to surface tension forces exerted during processing, resulting in partial loss of ordered arrangement. In all, we believe that these patterns are sufficient to demonstrate the versatility in nanoscale Si pattern generation of our approach and may be employed for a myriad of applications including nanoscale field effect transistors [1-3], biological, and chemical sensing [8], electrodes in Li-ion batteries [10], and nanocapacitor arrays [11].

Our work provides evidence of the controllability of the ordering, shapes, and dimensions of MCEE nanostructures by nanoimprinting, and general anisotropy in MCEE profiles simply by appropriate substrate orientation selection, mask material selection and connectivity of the catalytic layer. Further, by taking advantage of the fact that NIL moulds can be written with arbitrary patterns not necessarily of simple regular or periodic designs, we posit that complex three-dimensional nanostructures [35,36] with applications in photonics and optoelectronics can similarly be generated on a manufacturing scale for widespread implementation. In fact, through SRNIL, the patterns can be varied across the wafer by employing differently patterned moulds. Other nanoscale patterning techniques, for instance, interference lithography, and short-range self-assembly methods like AAO patterning, block copolymer, and nanosphere lithography are limited to producing periodic arrays of rod or wire-like shapes. Parallel and large-area wafer-scale patterning, as well as repeated use of a single mould, is further afforded by SRNIL. These features make our approach of SRNIL with MCEE more practically useful than other approaches published previously. The realization of long-range ordering of high aspect ratio Si nanostructures at sub-50-nm resolution with the aforementioned pattern versatility and on a wafer scale has not yet been reported.

## Conclusions

In conclusion, we demonstrate the versatile pattern generation of wafer-scale, highly uniform, well-ordered Si nanostructures with sub-50-nm resolution using a combination of step-and-repeat nanoimprint lithography and metal-catalyzed electroless etching. The long-range order and variability of nanoscale patterning offered in this approach cannot be achieved by self-organized methods of nanopatterning such as AAO templating, nanosphere lithography, and block copolymer self-assembly. Versatility in nanoimprint mould patterns allows this combinatory method to overcome the shortcomings of interference lithography and yet produce nanoscale features, previously limited to research-scale E-beam lithography or deep UV photolithography, on a wafer scale. The Si nanostructures produced in our approach show a high degree of fidelity as the user-defined SRNIL patterns, and retain non-porous top surfaces due to the substrate adherent, and chemically resistant SRNIL resin mask. This method is capable of producing high aspect ratio structures through a simple inexpensive wet etching setup. Minor lateral sidewall etching which arises from prolonged immersion in the etching solution reduces the dimensions of the Si nanostructures and should be taken into account in the design and fabrication process. Bearing these in mind, our approach could be very useful for large-scale nanostructured device production.

## Abbreviations

AAO: Anodic aluminum oxide; EBL: Electron beam lithography; IL: Interference lithography; MACE: Metal-assisted chemical etching; MCEE: Metal-catalyzed electroless etching; NIL: Nanoimprint lithography; RIE: Reactive ion etching; SAED: Selected area electron diffraction; SEM: Scanning electron microscopy; SiNWs: Si nanowires; SRNIL: Step-and-repeat nanoimprint lithography; TEM: Transmission electron microscopy.

## Competing interests

The authors declare that they have no competing interests.

## Authors’ contributions

JH conceived the idea and planned the experiments. JH and JD performed, analyzed, and optimized the step-and-repeat nanoimprint lithography process. JH performed the gold-assisted chemical etching and SEM. JH and QW carried out the TEM and analyzed the data. AT and SC participated in the design and coordination of the study. All the authors contributed to the preparation and revision of the manuscript, as well as, read and approved it.

## Authors’ information

JH and QW are Ph.D. candidates working on nanopatterning, fabrication, and growth of semiconductor nanostructures for photovoltaic and light-emission applications with the National University of Singapore (NUS). JD works on nanolithography and is with the Institute of Materials Research and Engineering (IMRE) of the Agency of Science, Technology and Research (A*STAR) in Singapore. AT is a Professor at the Department of Mechanical Engineering, NUS. SC is a Professor at the Department of Electrical and Computer Engineering, NUS.
